# Quantitative and ultrasensitive in situ immunoassay technology for SARS-CoV-2 detection in saliva

**DOI:** 10.1126/sciadv.abn3481

**Published:** 2022-05-25

**Authors:** Yuchao Chen, Fei Liu, Luke P. Lee

**Affiliations:** 1 WellSIM Biomedical Technologies Inc., 626 Bancroft Way, Suite A, Berkeley, CA, USA.; 2 Harvard Medical School, Department of Medicine, Brigham and Women’s Hospital, Boston, MA, USA.; 3 Department of Bioengineering and Department of Electrical Engineering and Computer Science, University of California at Berkeley, Berkeley, CA, USA.; 4 Institute of Quantum Biophysics, Department of Biophysics, Sungkyunkwan University, Suwon, Gyeonggi-do, Korea.

## Abstract

The coronavirus disease 2019 (COVID-19) pandemic has become an immense global health crisis. However, the lack of efficient and sensitive on-site testing methods limits early detection for timely isolation and intervention. Here, we present a quantitative and ultrasensitive in situ immunoassay technology for severe acute respiratory syndrome coronavirus 2 (SARS-CoV-2) detection in saliva (QUIT SARS-CoV-2). Our nanoporous membrane resonator generates a rapid oscillating flow to purify and concentrate fully intact SARS-CoV-2 virus in saliva by 40-fold for in situ detection of viral antigens based on chemiluminescent immunoassay within 20 min. This method can not only achieve a detection sensitivity below 100 copies/ml of virus, comparable to the bench-top PCR equipment; it can also improve detection specificity via direct monitoring of viral loads. The integrated portable QUIT SARS-CoV-2 system, which enables rapid and accurate on-site viral screening with a high-throughput sample pooling strategy, can be performed in primary care settings and substantially improve the detection and prevention of COVID-19.

## Teaser

Integrated sample preparation and quantitative immunoassay enables rapid, ultrasensitive detection of SARS-CoV-2 virus in saliva.

## INTRODUCTION

Currently, the emergence of the novel coronavirus, especially the Delta and Omicron variants, is rapidly spreading across the world, causing massive health care burdens and economic shutdowns at an unprecedented scale (*1*, *2*). Unfortunately, the daily reported positive cases and deaths continue to grow. One of the reasons is the limitations of existing testing capabilities, including a scarcity of convenient tests, time-consuming workflows, and the requirement for samples to be processed in a laboratory with specialized equipment for sensitive molecular diagnostic methods.

Most current molecular diagnostic technologies rely heavily on polymerase chain reaction (PCR), isothermal nucleic acid (NA) amplification, or sequencing-based methods that involve a multistep approach for sample preparation (*3*–*7*). While sensitive, the protocol is time-consuming, particularly if viral RNA extraction is required. Furthermore, these medium- or high-complexity tests require highly skilled laboratory technicians for operation. Another concern is that primers for different genes can be directly affected by variations in the genomic sequences of the virus due to their rapid mutations, which can generate false-negative results (*8*). Recently, the Food and Drug Administration announced that several reverse transcription PCR (RT-PCR)–based tests were unable to detect the severe acute respiratory syndrome coronavirus 2 (SARS-CoV-2) Omicron variant. Conversely, point mutations in the N protein are less likely to occur, which is considered the best target for in vitro diagnostic detection because of the conservation of the N protein sequence (*9*).

Lateral flow assays (LFAs) that probe for the viral antigens or antibody response against the virus are also being actively developed (*10*, *11*). While allowing timely testing with low cost, these assays lack sensitivity and cannot detect infection at its early stage, when the infected subjects can already transmit virus (*12*). To limit the virus spread, rapid, sensitive, and convenient diagnostic tests are required to identify a person’s infection status as early as possible. A cost-effective technology that permits rapid on-site detection of the virus with high sensitivity and specificity, as well as minimum user intervention (e.g., little hands-on time and low risk), is highly desirable in the ongoing pandemic.

**Fig. 1.**
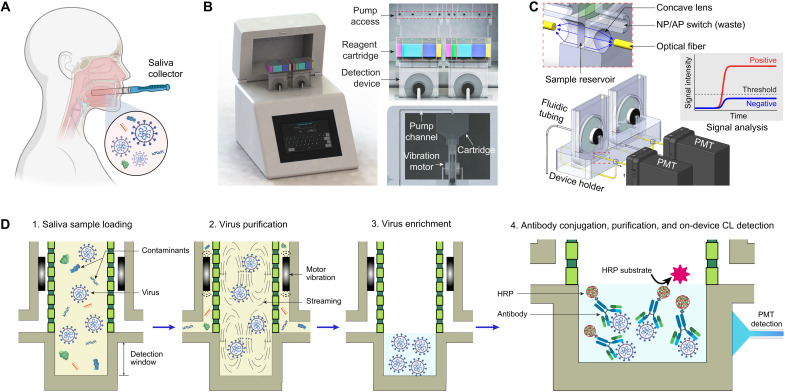
Illustration of the QUIT SARS-CoV-2 system and workflow. (**A**) Illustration showing saliva collection for SARS-CoV-2 virus detection. (**B**) Illustration of the QUIT SARS-CoV-2 system including a workstation and two disposable purification and detection devices. (**C**) Illustration of the detection module in a QUIT SARS-CoV-2 system. AP, air pressure; NP, negative pressure; PMT, photomultiplier tube. (**D**) Illustration showing the workflow for virus purification, enrichment, and detection using the QUIT SARS-CoV-2 system. CL, chemiluminescence; HRP, horseradish peroxidase.

Here, we demonstrate a quantitative and ultrasensitive in situ immunoassay technology for SARS-CoV-2 detection in saliva (QUIT SARS-CoV-2). Instead of nasopharyngeal swab specimens, our approach uses raw saliva as a specimen (Fig. 1A), which not only is easier for collection but also shows higher detection sensitivity in the first 10 days after infection as reported by Wyllie *et al.* (*13*). In the study, they also detected more SARS-CoV-2 RNA copies with smaller variations in the saliva specimens than in the nasopharyngeal swab specimens of the same patients, indicating the reliability of saliva as an alternative diagnostic specimen. The QUIT SARS-CoV-2 system as shown in Fig. 1B comprises a workstation with two disposable QUIT SARS-CoV-2 devices in it, enabling analysis of two saliva samples simultaneously. The workstation was developed by integrating a detection module and a fluidic module for automatic system operation and actuation (Fig. 1C and fig. S1). Distinct from microfluidic approaches for nanoparticle separation (*14*–*18*), the QUIT SARS-CoV-2 devices integrate two nanoporous membrane resonators and a detection window into the sample reservoir, allowing rapid virus purification and concentration followed by high-sensitivity analyte detection. Two vibration motors attached to the nanoporous membranes and alternating negative pressure applied on the two outlets together limit the buildup of membrane fouling layers, which would otherwise clog the nanopores (*19*). Saliva is a medium with high viscosity, rich enzyme concentration, and wide variations, resulting in enormous challenges for sample preparation. With this design, the SARS-CoV-2 virus can be isolated from 2 ml of saliva samples and enriched by 40-fold into a final volume of 50 μl within 3 min, inherently enabling a high-throughput sample pooling approach (*20*, *21*). To minimize hands-on operation and prevent aerosol contamination, a disposable reagent cartridge and two disposable waste containers are integrated with the QUIT SARS-CoV-2 device for sequential injection of sample and reagents as well as the collection of liquid waste (Fig. 1B and fig. S2).

## RESULTS AND DISCUSSION

The workflow for virus enrichment and detection is shown in Fig. 1D. After loading 2 ml of raw saliva from the saliva collector into the reagent cartridge (fig. S3), the sample, mixed with 3 ml of prefilled diluent buffer, is injected into the isolation chamber of the system by applying a positive pressure onto the reagent cartridge. During this process, the saliva sample was prefiltered via a 600-nm nonporous membrane installed inside the reagent cartridge (fig. S2B) to remove large particles (e.g., cells and debris). Alternating negative pressure is then applied onto the two outlets to remove the molecular contaminants (e.g., proteins and NAs) from the 20-nm nanopores (Fig. 1D). The intact viruses are retained inside the isolation chamber due to their larger size (between 50 and 200 nm). The vibration motor–induced streaming inside the chamber helps to resuspend the viruses and other particles into the fluid, which can not only limit membrane clogging but also improve virus recovery. After draining the excess liquid, the sample is eventually enriched into 50 μl. The concentrated analyte is located inside the detection chamber at the bottom of the system. A mixture of Spike S1 and S2 antibodies labeled with horseradish peroxidase (HRP), the enzyme for chemiluminescent reaction, is then pumped into the system for bioconjugation with SARS-CoV-2 viruses by targeting their surface antigens. The antigen-antibody reaction is incubated at room temperature for 5 min before the washing buffer is injected, and the analyte is washed three times to get rid of the unconjugated antibodies and HRPs. During the incubation, the motors continue to vibrate to improve the molecular interactions. Eventually, the chemiluminescent substrate is injected into the system for chemiluminescent reaction under the catalyzation of HRP. The emitted luminescent signal for analysis was then detected by a photomultiplier tube (PMT) through an optical fiber aligned with the detection window of the system. The PMT converted the luminescent signals into amplified electrical signals, represented as relative light units (RLU), which was eventually recorded by a microcontroller as output.

**Fig. 2.**
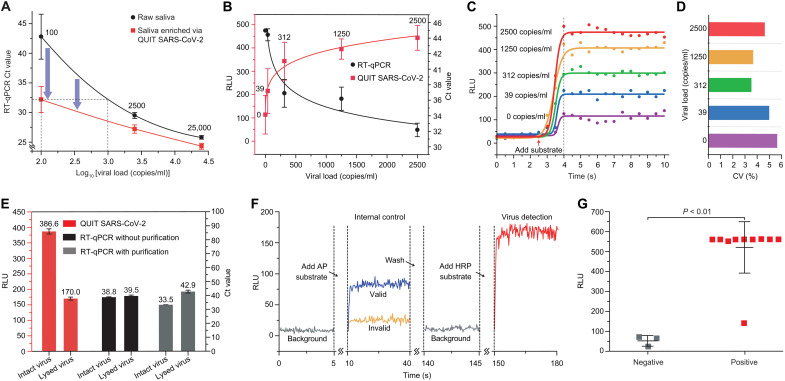
Validation and application of the QUIT SARS-CoV-2 system. (**A**) Comparison of RT-qPCR Ct values from samples with and without enrichment by the QUIT SARS-CoV-2 system. SARS-CoV-2 viruses spiked into pooled saliva from healthy donors were used as study samples. (**B**) Characterization of QUIT SARS-CoV-2 using pooled saliva samples spiked with SARS-CoV-2 virus at different concentrations (red curve present as RLU values). RT-qPCR was carried out for comparison (black curve present as Ct values). Ct value was recorded as 45 when no signal was detected. Pooled saliva without spiked virus (0 copies/ml) was used as negative controls. (**C**) Chemiluminescent signals detected by a PMT at different viral loads. The curves were fitted by the sigmoid function. (**D**) Coefficient of variation (CV) for the signal intensities on the plateau collected in 30 s. (**E**) Comparison of the detection results for intact virus and lysed virus after enrichment by the QUIT SARS-CoV-2 system. Chemiluminescent detection by QUIT SARS-CoV-2 and RT-qPCR was used for the characterization of viral antigen and RNA. (**F**) Illustration showing the luminescent signals detected by QUIT SARS-CoV-2 including the detection of internal control and virus. AP, alkaline phosphatase. (**G**) Application of QUIT SARS-CoV-2 in the study of COVID-19 patient samples and healthy controls. Mann-Whitney test was used to calculate the *P* value. RLU, relative light unit.

To validate the system’s performance, we first studied its capability of virus purification and enrichment by concentrating pooled saliva samples with three viral loads (i.e., 25,000, 2500, and 100 copies/ml) for RT-quantitative PCR (RT-qPCR) analysis. For each viral load concentration, three sets of aliquots were used. We enriched 800 μl of each sample into a final volume of 60 μl, which theoretically has a 13.3-fold concentration improvement. Compared to the original saliva samples before enrichment, all the samples processed via QUIT SARS-CoV-2 had even lower cycle threshold (Ct) values as shown in Fig. 2A, indicating a higher detection sensitivity. Especially for the samples with a lower virus concentration, a higher increase in sensitivity was observed. At the concentration of 100 copies/ml, the averaged Ct value was 32.2 after enrichment, while in two of the three original samples, no signal was detected within 45 cycles. Since the viral load of 100 copies/ml in raw saliva was beyond the detection limit of RT-qPCR, the detection sensitivity had an exponential enhancement after enrichment (i.e., more than 1000-fold). The RT-qPCR analysis demonstrated that our system could effectively concentrate the SARS-CoV-2 virus and improve the detection limit. In our integrated on-chip isolation and detection assay, 40-fold enrichment could be achieved for higher sensitivity improvement.

The QUIT SARS-CoV-2 system was then used to enrich and analyze 15 blind saliva samples with five different viral loads from 0 (negative control) to 2500 copies/ml and compared to RT-qPCR as shown in Fig. 2B. The chemiluminescent reaction emits strong optical signals as soon as the HRP substrate is added and maintains the signal intensities on a stable level for minutes (Fig. 2C). After the signal intensity reached a plateau, the coefficient of variation (CV) was generally below 10% without substantial fluctuation (Fig. 2D). Therefore, the QUIT SARS-CoV-2 system collected the luminescent signals on the plateau for 30 s to obtain an average detection signal intensity. As the viral load decreased from 2500 to 0 copy/ml, the signal intensities also gradually decreased. The signals detected from the positive samples, even at as low as 39 copies/ml, could be distinguished from the negative control, rivaling the sensitivity of the RT-qPCR method (Fig. 2B). Above the concentration of 312 copies/ml, the signal intensities of all replications had no overlap with the negative controls, which could be identified as positive results.

Although detection sensitivity usually draws more attention than specificity, more and more concerns have been raised regarding the false-positive testing results (*22*–*24*). Even a very small false-positive rate (e.g., <1%) may result in a substantial proportion of false-positive results, especially when the prevalence of the virus in the population is low (*25*). One unique feature of our QUIT SARS-CoV-2 is that it only isolates and analyzes intact virus particles while getting rid of most viral debris, which otherwise can still be detected from a large proportion of recovered patients (*26*). We prepared two aliquots of viral specimens with one of them lysed before the study. The samples were purified and concentrated by the QUIT SARS-CoV-2 system followed by chemiluminescent and RT-qPCR detection of antigen and RNA, respectively (Fig. 2E). In both analyses, the lysed virus’ signal intensities were significantly lower compared to the signal from the intact virus, indicating that viral debris was substantially removed after purification by QUIT SARS-CoV-2. The viral specimens, lysed via heating or enzyme digestion, may still contain intact virus particles, resulting in residual signals being detected. Further experiments, such as inspection via an electron microscope, need to be carried out to verify this observation. To the best of our knowledge, among the existing techniques, QUIT SARS-CoV-2 is the only testing that detects fully intact virus particles, allowing for direct monitoring of viral loads. Studies have revealed that viral infectivity and transmission potential are highly correlated with the viral load in relevant clinical sites (*27*). Therefore, our technique will be able to evaluate the transmission risk at a certain instant with much higher accuracy than the conventional methods [e.g., RT-PCR, enzyme-linked immunosorbent assay (ELISA), and LFA]. Furthermore, the concentration of spike proteins could be derived from viral load and RNA quantity measured by QUIT SARS-CoV-2 and RT-qPCR, respectively, which will facilitate our understanding of the necessary sensitivity and specificity of the common antigen tests.

An internal control system was developed and implemented by using inactivated Influenza A H1N1 virus and alkaline phosphatase (AP)–labeled anti-hemagglutinin (HA) antibody to ensure detection accuracy. The detection process is shown in Fig. 2F. After sample purification and concentration, AP substrate was first added for detection of the internal control. The sample was then washed once to stop light emission and remove the background signal before HRP substrate was added for SARS-CoV-2 virus detection. Only when the signal intensity of internal control was higher than a certain level (i.e., >50 RLU) was the test considered valid.

Eventually, we applied the QUIT SARS-CoV-2 system in the analysis of individual saliva samples from 10 patients with coronavirus disease 2019 (COVID-19) and three healthy controls (Fig. 2G). All the patient samples had Ct values between 18 and 23 analyzed by RT-qPCR (table S1). Our study found that most of the patient samples emitted much stronger luminescent signals (>10-fold) than the healthy controls, even exceeding the PMT’s output saturation level at 560 RLU. Further integration of a less sensitive optical sensor will solve this issue and obtain linear signal enhancement as virus concentration increases. Although one patient sample, which may experience degradation after melting overnight, was detected with a lower signal intensity at around 140 RLU, it was still differentiated from the negative samples with an RLU below 100. We performed receiver operator characteristic analysis of the testing results, which indicated an RLU cutoff value of 106.03, twice of the average RLU of negative controls. With this cutoff value, the QUIT SARS-CoV-2 system can successfully identify all the patient samples in the study. We further performed Fisher’s exact test to determine the relationship between testing results of RT-PCR and QUIT SARS-CoV-2 with a *P* value (0.0035) smaller than 0.05, indicating that there is a significant relationship between RT-PCR examination and our method. Although promising, the study of clinical samples via the QUIT SARS-CoV-2 system was preliminary and limited to a small sample size and high viral load of patient samples (18.5 to 23.1 of the Ct values). To further verify this technology in virus detection, a larger number of patient samples with lower viral loads and healthy controls need to be tested to ensure the sensitivity and specificity, as well as to optimize the RLU cutoff value in the future.

In summary, we have demonstrated a quantitative and ultrasensitive in situ immunoassay to detect the SARS-CoV-2 virus in saliva economically, which costs less than $1000 for one instrument and less than $15 for each testing. Compared to the NA detection methods (*28*–*30*) and other antigen detection approaches such as LFAs and ELISA (*10*–*12*), our technology offers a monolithic and elegant solution for unmet needs with multiple unique advantages (Table 1). First, it is the first nanoporous membrane device with fast purification due to the harmonic oscillator’s vibratory shear-enhanced process and high shear cleaning action. Our system allows us to accomplish 40× sample enrichment and highly sensitive chemiluminescent immunoassay in an isolated chamber, enabling rapid sample-to-answer on-site testing (<20 min) with PCR-level detection sensitivity (as little as 39 copies/ml of viral load). Because of its high sensitivity and large sample volume load (i.e., 2 ml of raw saliva), a sample pooling strategy (e.g., twenty 100-μl saliva samples per pool) can be seamlessly implemented for a high-throughput manner without the need for additional equipment. Implementation of a prescreening test with the QUIT SARS-CoV-2 system would be helpful to reduce workload and cost of RT-PCR test for each individual specimen. Second, our method could improve detection specificity with a more accurate evaluation of the transmission risk by isolating and detecting only intact virus particles while getting rid of viral debris and other molecular contaminants, which otherwise can potentially cause false-positive testing results. Third, the QUIT SARS-CoV-2 system uses a non-invasive method for sample (saliva specimens) collection and virus detection. This method not only could simplify sample collection and minimize interaction between health care providers and potentially infected individuals but also has higher detection sensitivity in the early-stage infection, which could become a practical solution for detection of the rapidly spreading Delta and Omicron variants (*13*, *31*). With these features, the QUIT SARS-CoV-2 system is ideal for on-site screening testing (e.g., at airports, schools, communities, and outpatient clinics), facilitating timely identification of suspected virus infection.

**Table 1.** Comparison of different detection methods for SARS-CoV-2. LOD, limit of detection; POCT, point-of-care testing.

**Method**	**Target**	**LOD (copies/ml)**	**Lead time**	**Cost per sample**	**POCT**
**RT-qPCR**	RNA	10^2^	2–5 hours	<$1	No
**LFA**	Antigen	10^6^	~20 min	<$1	Yes
**QUIT**	Virus particle	10^2^	~20 min	<$1*	Yes

*Based on pooled sample testing.

## MATERIALS AND METHODS

### QUIT SARS-CoV-2 device

The disposable device for SARS-CoV-2 virus isolation and detection was fabricated by assembling two symmetrical structures. Two optically transparent polymethyl methacrylate (PMMA) parts were manufactured via computer numerical control milling, with anodic aluminum oxide (AAO) membrane (20-nm pore size, 25 mm diameter; WHA68096002, Sigma-Aldrich) attached on each part using epoxy adhesive. A vibration motor (1597-1244-ND, Digi-Key) was immobilized between the AAO membrane and the PMMA structure. Its electric wires are connected to a 3-V DC power supply through an opening on the PMMA structure during operation. The two symmetrical parts were assembled using epoxy adhesive to prevent liquid leakage. The disposable reagent cartridge and waste containers were fabricated via stereolithography and integrated with the QUIT SARS-CoV-2 device (fig. S2A). The reagent cartridge was connected to the system inlet for reagent injection, while the two waste containers were connected to the two system outlets for waste collection. The reagent cartridge, with a polycarbonate track etch nanoporous membrane (600 nm; PCT0647100, Sterlitech) sealed at the outlet, had six chambers for storage of different reagents including washing buffer, diluent buffer for the saliva sample, antibodies, AP substrate, HRP substrate, and peroxide solution (fig. S2B).

### QUIT SARS-CoV-2 workstation

The internal configuration of the workstation is illustrated in fig. S1B. The instrument enclosure was manufactured via selective laser sintering. A microcontroller (Mega 2560, Arduino) was programmed to actuate the system and collect luminescent signals automatically. A diaphragm pump applied 15-kPa positive pressure to the reagent cartridge through a valve group to sequentially inject sample and reagents into the virus isolation chamber. Another diaphragm pump applied 15-kPa negative pressure to the two openings of the waste containers through a pair of three-way valves to collect liquid waste from the isolation chamber. To enable on-site testing, no liquid was circulated inside the workstation. By controlling the two valves, the negative pressure was periodically applied to one waste container every 15 s, with another one exposed to the air pressure to achieve negative pressure oscillation. The optical signals emitted from the chemiluminescent reaction were detected by a PMT (H10721-01, Hamamatsu) via an optical fiber (BF23P, Banner) aligned to the detection window on the system. The PMT converted the optical signals into electrical signals, collected by the microcontroller through a transimpedance amplifier and a voltage divider. It was then recorded by the Arduino IDE software (version 1.8.13) for analysis.

### System operation

After plugging the QUIT SARS-CoV-2 device into the workstation, 2 ml of raw saliva from a saliva collector was injected into the reagent cartridge, followed by connection of gas tubing with the cartridge for sequential sample and reagent injection. The cover of the workstation was then closed to limit background signal of PMTs. After turning on the power and running the script via a laptop connected to the workstation, the sample was processed automatically for around 20 min with electrical signals displayed on the laptop. The QUIT SARS-CoV-2 device was disposed after use.

### Reagents for virus enrichment and chemiluminescent immunoassay

For system characterization, inactivated SARS-CoV-2 viruses [50,000 copies/ml; NATSARS(COV2)-ERC, ZeptoMetrix] were spiked into pooled saliva (991-05-P-250, Lee Biosolutions) as testing samples. Individual saliva samples from patients with COVID-19 and healthy controls were purchased from AMSBIO stored at −20°C until testing. Clinical materials are obtained following official protocols, with appropriate Institutional Review Board/Independent Ethics Committee approval. Saliva samples (2 ml) were used for testing in each run unless otherwise noted. A saliva collection and purification system (Pure-SAL, Oasis Diagnostics) was used to transfer the saliva sample to the reagent cartridge (fig. S3). A 1× tris-buffered saline (TBS) buffer (T0537, TEKnova) containing 0.05% Tween 20 (T0710, TEKnova) was used as the washing buffer (25 ml) and diluent buffer (3 ml). Inactivated Influenza A H1N1 virus (100 μl; NATFLUAH1N1-ERCM, ZeptoMetrix) was added into the diluent buffer as an internal control. To prepare the antibody mixture, SARS-CoV-2 Spike S1 antibody (GTX635654, GeneTex) and Spike S2 antibody (GTX632604, GeneTex) as well as Influenza A virus H1N1 hemagglutinin (HA) antibody (GTX127357, GeneTex) were biotinylated via a biotinylation kit (ab201795, Abcam) as per the manufacturer’s protocol. The S1 and S2 antibodies were labeled with streptavidin-conjugated HRP (21130, Thermo Fisher Scientific). In contrast, the HA antibody was labeled with streptavidin-conjugated AP (434322, Thermo Fisher Scientific). Each antibody (1 μl) was then mixed and diluted into 100 μl by 1× TBS buffer as an antibody solution. HRP substrate (100 μl; A38554, Thermo Fisher Scientific) and AP substrate (100 μl; WP20002, Thermo Fisher Scientific) were used for chemiluminescent reaction to detect SARS-CoV-2 virus and internal control.

### RT–quantitative polymerase chain reaction

RT-qPCR was performed following an NA extraction-free approach, in which 50 μl of virus sample was first treated with 6.25 μl of proteinase K followed by a heat inactivation step and was then directly used as input. The 2019-nCov CDC EUA Kit (10006770, Integrated DNA Technologies) for detection of N1, N2, and ribonuclease P genes and a Reliance 1 step multiplex enzyme supermix (12010176, Bio-Rad) were used to prepare PCR cocktail including 1 μl of N1 probe, 1 μl of N2 probe, 6.25 μl of enzyme mix, 1.75 μl of nuclease-free water, and 15 μl of proteinase K–treated virus sample. The RT-qPCR was conducted on a Real-Time PCR Detection System (CFX384, Bio-Rad) following the protocol: 52°C for 10 min, 95°C for 2 min, and 45 cycles of 95°C for 10 s and 55°C for 30 s. The Ct value for the samples without detection signal was recorded as 45 for quantitative comparison. To obtain the lysed virus sample, viruses were incubated at 65°C for 30 min for antigen analysis by the QUIT SARS-CoV-2 system and treated with proteinase K for 5 min for NA analysis by RT-qPCR.

### Statistical analysis

All the experiments in the study were repeated at least three times except for the analysis of clinical samples. Origin 9.1 software was used for graphical representation and statistical analyses. The error bars in the graphical data represent the means ± SDs. Statistical significance was determined using a Mann-Whitney test. A *P* value of <0.05 indicates statistical significance. Fisher’s exact test was used to analyze nonrandom associations between the testing results of RT-PCR and QUIT SARS-CoV-2. The relationship is significant at *P* < 0.05.

## Notes

View/request a protocol for this paper from *Bio-protocol*.
