# Supplementary material for: Quantitative and ultrasensitive in situ immunoassay technology for SARS-CoV-2 detection in saliva
Source: Sci Adv. 2022 May 25;8(21):eabn3481. doi: 10.1126/sciadv.abn3481 (PMC9132547; doi:10.1126/sciadv.abn3481)
Supplement: Supplementary file 1 — Figs. S1 to S3 Table S1 [file sciadv.abn3481_sm.pdf]

Science Advances
AAAS

Supplementary Materials for

Quantitative and ultrasensitive in situ immunoassay technology for SARS-CoV-2 detection in saliva

Yuchao Chen *et al.*

Corresponding author: Fei Liu, fliu2080@gmail.com; Luke P. Lee, lplee@bwh.harvard.edu

Sci. Adv. **8**, eabn3481 (2022)
DOI: 10.1126/sciadv.abn3481

This PDF file includes:

Figs. S1 to S3
Table S1

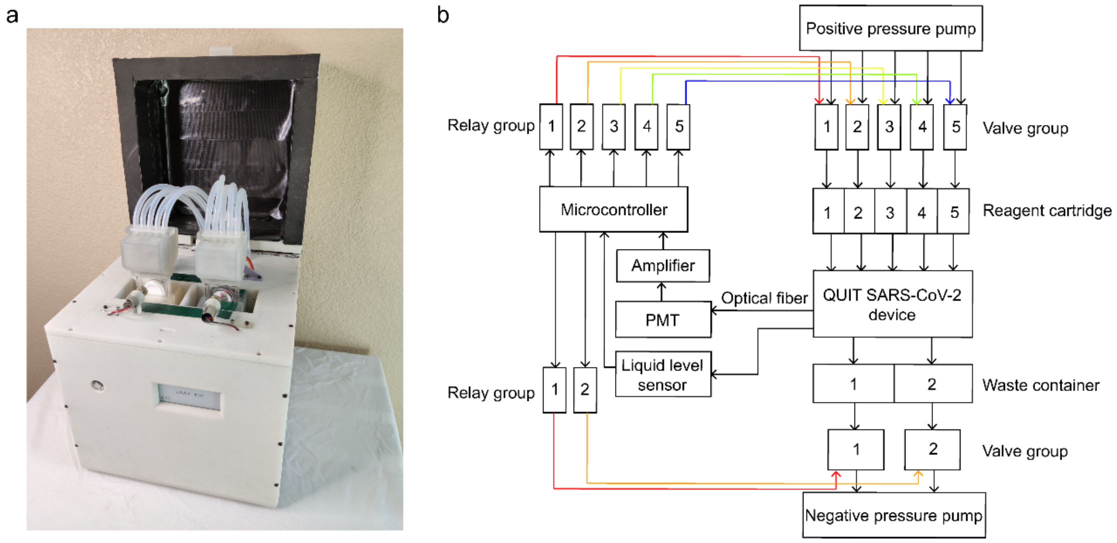

Figure S1. (**a**) Image of a prototype of the QUIT SARS-CoV-2 system. Each system can run two samples simultaneously. (**b**) Illustration showing the working principle of the QUIT SARS-CoV-2 system.

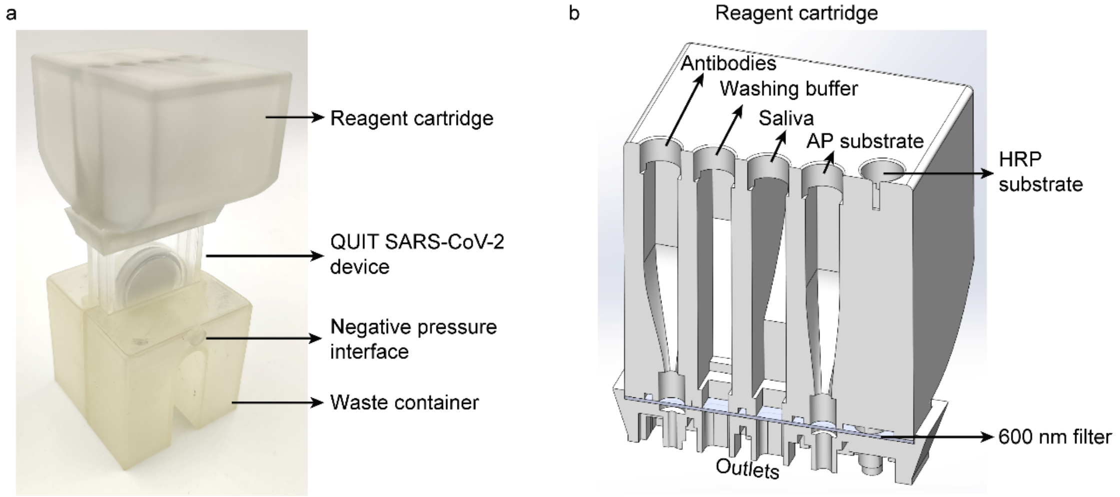

Figure S2. (**a**) Image of the disposable components including reagent cartridge, QUIT SARS-CoV-2 device, and waste containers for point-of-care testing. (**b**) Cross-section view of the reagent cartridge design.

a b c

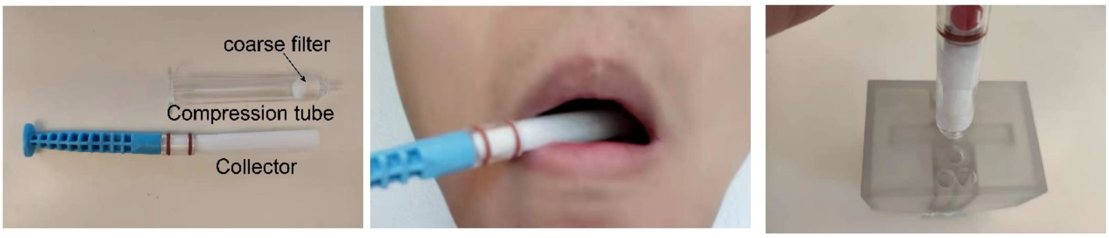

Figure S3. (**a**) An image showing the saliva collector including a sponge collector and a compression tube. (**b**) Collect 2 mL of saliva by placing the absorbent sponge into mouth for ~60 s. (**c**) Inject the saliva sample into the reagent cartridge by compressing the absorbent sponge.

Table S1. Information of COVID-19 patients and healthy controls in our study.

ID	Age	Gender	Individual	Date of test	Ct Value	RLU value
1	75	M	Patient	2020/9/10	21.00	140.7
2	58	M	Patient	2020/9/9	19.40	560.1
3	57	M	Patient	2020/9/10	22.00	560.0
4	40	M	Patient	2020/9/11	21.10	551.2
5	48	F	Patient	2020/9/10	20.50	560.0
6	50	F	Patient	2020/9/10	18.70	560.6
7	62	F	Patient	2020/9/12	23.10	560.1
8	63	F	Patient	2020/9/10	22.60	561.9
9	24	M	Patient	2020/9/9	18.50	560.4
10	51	M	Patient	2020/9/10	23.00	560.3
11	50	M	Control	2020/9/10		23.8
12	38	F	Control	2020/9/9		64.7
13	34	M	Control	2020/9/10		71.4
